# Supplementary material for: Novel Sustained-Release Drug Delivery System for Dry Eye Therapy by Rebamipide Nanoparticles
Source: Pharmaceutics. 2020 Feb 14;12(2):155. doi: 10.3390/pharmaceutics12020155 (PMC7076486; doi:10.3390/pharmaceutics12020155)
Supplement: Supplementary file 1 [file pharmaceutics-12-00155-s001.pdf]

pharmaceutics

Supplementary Materials: Novel Sustained-Release Drug Delivery System for Dry Eye Therapy by Rebamipide Nanoparticles

Noriaki Nagai, Miyu Ishii, Ryotaro Seiriki, Fumihiko Ogata, Hiroko Otake, Yosuke Nakazawa, Norio Okamoto, Kazutaka Kanai, and Naohito Kawasaki

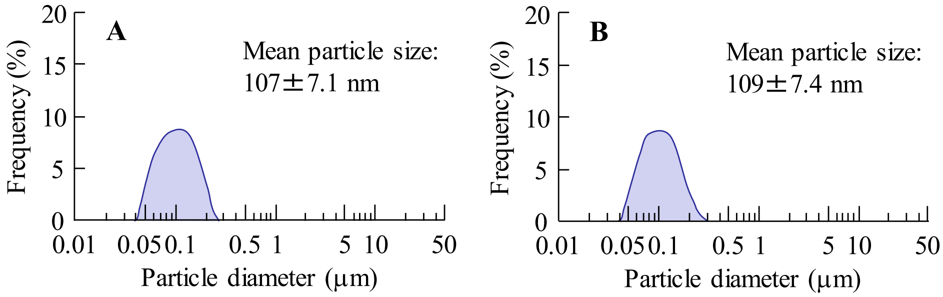

Figure S1. Particle size distribution of rebamipide solid in REB-NPs on the day of (**A**) and 1 month after (**B**) preparation. The data were obtained by the laser diffraction measurement.

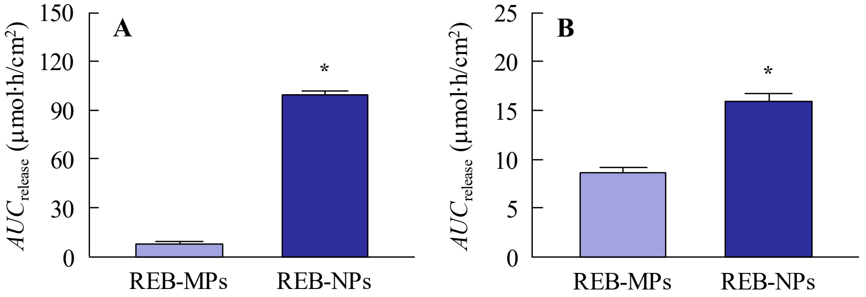

Figure S2. $AUC_{release}$ for REB-MPs and REB-NPs through the 25 nm (**A**) and 450 nm (**B**) pore membranes. n = 5–6. *$P < 0.05$, vs. REB-MPs.

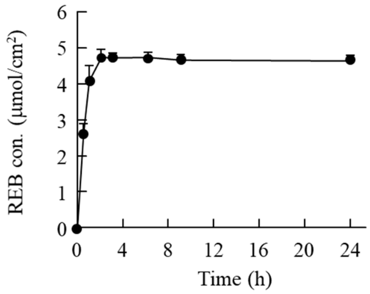

Figure S3. Drug release from REB formulations containing dissolved rebamipide through 450 nm pore membranes. The N,N-dimethylformamide was used to dissolve the rebamipide. n = 4.

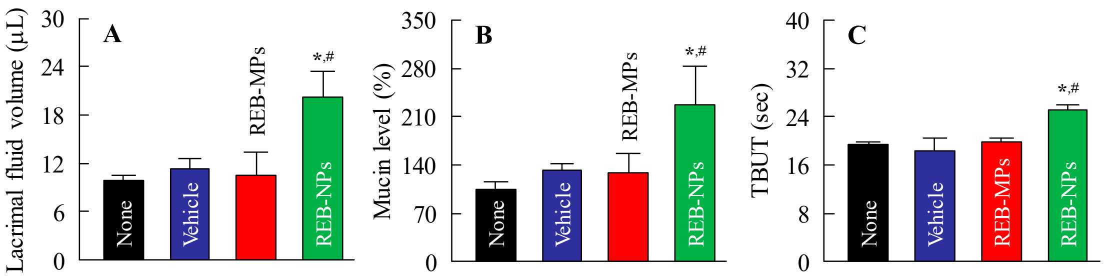

Figure S4. Lacrimal fluid volume (**A**), mucin levels (**B**) and TBUT (**C**) in normal rabbits single applied with REB formulations. The REB formulations were applied for 6 h. *$P < 0.05$, vs. None for each group. #$P < 0.05$, vs. Vehicle for each group.
